# On Interesting Cases

**Published:** 1907-08-17

**Authors:** 


### Resident Medical Officers' Department.

[Contributions to this Column are invited, and, if accepted, will be paid for.]

### ON INTERESTING CASES.

The resident medical officers in a great hospital are supposed to represent the finished products of modern scientific medical education. During their term of office, as a natural result of their upbringing, they desire, above all things, to have under their care a large proportion of interesting cases, and in this they are encouraged by their superior officers. The category of interesting cases we may take to include two distinct classes of patients.

Firstly, there are the patients suffering from acute diseases, such as enteric fever, appendix abscess, lobar pneumonia, strangulated hernia, pericarditis, and so forth. Although comparatively common in hospital practice, no two of these cases are exactly alike, and each is full of dangerous and interesting possibilities. No resident would dream of sending away a patient afflicted with one of these severe conditions, even if it were necessary to put a convalescent patient on a mattress on the ward floor for the night, in order to take him in. Every case of this description requires instant admission so soon as its gravity is recognised, whether or no a definite diagnosis can at first be made. The surgical emergencies usually require immediate operation; while those relegated to the medical side of the hospital are always liable to present alarming symptoms which call for summary treatment. Acute diseases such as these are full of interest, and provide ample opportunities for the display of courage, imperturbability and resource. Their recovery affords, perhaps, the keenest satisfaction that the house officer can experience. To the nursing staff they are the cases above all others which offer the best field for the display of skill and devotion.

The second group of cases, which the house officer admits because they are interesting, includes all those unfortunates whose bodies are little less than pathological curiosities. These patients seldom afford any scope for successful treatment beyond the temporary relief of symptoms; their claim to admission into the wards is based upon the faithfulness with which they follow the text-book descriptions of rare and interesting conditions. Thus the great majority of diseases of the central nervous system are of diagnostic interest only; their victims are merely living museums, the despair of therapeutists. Beyond the fact that rest in hospital and nursing by skilled hands is some amelioration of their misery, these patients are of about as much practical use to the student of the healing art as the acrostic pages of a lay periodical. They have really no more right to the coveted beds of general hospitals than the so-called dull cases of chronic heart or joint or kidney disease, which are often turned off to the infirmary. The clinical study of recondite central nervous diseases, so dear to the heart of the ultra-scientific physician, is of academic interest only. A vague recollection of the symptoms and morbid appearances of these rare and obscure lesions may even be an embarrassment rather than a help to the busy general practitioner.

We thus come to this conclusion, that the first group of interesting cases, comprising the acute diseases usually amenable to treatment, not only demands instant admission, but is invariably a source of profit to the resident medical officer, both from the point of view of diagnosis and of treatment; while the usual course of procedure which rejects whenever possible cases of common chronic disorders, and admits in their stead examples of rare and remote lesions inaccessible to treatment, is short-sighted and illogical.

The best that can be said of extended investigations into obscure neurological problems is that they encourage habits of close observation, accurate description, and careful sifting of scattered items of evidence. At the same time they recall the details of physiology and anatomy—gross and minute—and enliven them with a new interest and a new significance. But nearly all these elaborate investigations, which, in our opinion, are very much overdone, can be carried out as well in the out-patient rooms as in the wards, with equal educational advantage to the students and almost equal benefit to the patients. Whereas the despised and rejected osteoarthritis, the chronic nephritis, water-logged and tedious, the dull bronchitic veteran, and the moribund victim of phthisis, are clinical material worthy of something better than contemptuous diagnosis, and routine out-patient treatment with drugs, or hasty dismissal to the infirmary.

